# Evaluation of the effectiveness of osteopathic treatment on the mother-newborn dyad in the event of painful breastfeeding in a maternity hospital despite the application of all usual aids: randomized interventional trial in two parallel arms without blinding

**DOI:** 10.3389/fnut.2025.1577502

**Published:** 2025-04-24

**Authors:** Christophe Elleau, Corine Missmahl, Wafae Belcadi, Valérie Aurillac-Lavignolle, Loic Sentilhes

**Affiliations:** ^1^Néonatalogie A, Pôle Pédiatrie, Hôpital Pellegrin, Centre Hospitalier Universitaire de Bordeaux, Bordeaux, France; ^2^Centre Hospitalier Universitaire de Bordeaux, Département de Gynécologie-Obstétrique, Bordeaux, France; ^3^Centre Hospitalier Universitaire de Bordeaux, CIC, Hôpital Pellegrin, Bordeaux, France

**Keywords:** breastfeeding, pain, painful breastfeeding, lactation duration, osteopathic manipulative treatment

## Abstract

**Aim:**

Pain during breastfeeding is the first reason for stopping breastfeeding. When conventional aids are ineffective, osteopathic manipulative treatment (OMT) may be an option to reduce pain and improve the quality and duration of breastfeeding. The objective is to compare conventional aids (untreated group) versus conventional aids combined with OMT (tactile listening) for both the baby and the mother (treated group). The primary outcome is the breastfeeding rate 1 month after birth. Patients were included in the maternity ward of the University Hospital Center of Bordeaux from March 23, 2022, to April 23, 2024. The inclusion criteria was breastfeeding pain greater than 7 (scale from 0 to 10) in one or both breasts. The mother had to be aged 18 years or older, with a singleton newborn, born after 37 weeks of gestation, weighing at least 2,500 g and included after 36 h of life.

**Results:**

23 mother-infant dyads were included, 13 in the treated group and 10 in the untreated group. The average pain score for the most painful breast was 9/10 in both groups. One month after birth, breastfeeding was ongoing in 11 dyads in the treated group and 3 in the untreated group (*p* = 0.01), exclusive breastfeeding was ongoing in 8 dyads in the treated group and 1 in the untreated group (*p* = 0.03). Three dyads left the study after stopping breastfeeding.

**Discussion:**

Our study was prematurely terminated due to difficulties in patient recruitment caused by breastfeeding cessation or parental refusal of randomization. OMT appears useful in cases of highly painful breastfeeding, facilitating the effectiveness of professionals providing conventional support. Another study conducted in France did not show a beneficial effect: only the baby was treated with the mother separated behind a screen, the inclusion criteria was composite (IBFAT), OMT was not clearly defined as soft touch. Treating the baby without the mother is not relevant, particularly when the objective is to improve breastfeeding, which requires perfect synchrony between mother and infant. We consider it crucial to conduct further studies, enrolling participants earlier and targeting an audience without prior knowledge or biases regarding osteopathy.

**Clinical trial registration:**

http://www.clinicaltrials.gov/, identifier AMATOSTEO NCT 05185323.

## 1 Introduction

The practice of breastfeeding is challenging due to the loss of intergenerational knowledge transfer and the influence of several factors that shorten its duration. The primary reason for cessation is pain, followed by the mother’s return to work (1).

Currently, more than 80% of women express the desire to breastfeed, but breast pain leads to frequent cessation, particularly before hospital discharge. Nipple pain arises from various factors: excessive pressure from a tense newborn, overly vigorous sucking, poor positioning of the newborn and/or the mother due to discomfort or stress. To improve oral functionality (2, 3) and reduce stress levels for both the newborn and/or the mother (4), it appears necessary to address them simultaneously. When medical and paramedical interventions fail to resolve such pain, osteopathy may offer a potential solution.

Osteopathy remains poorly studied: in 2012, reports from INSERM (5) and Assistance Publique des Hôpitaux de Paris (6) highlighted the need for further evaluation. The latest research on osteopathy in perinatology and pediatrics (4) emphasizes that while most studies to date suggest a benefit, the evidence level remains low, except regarding reduced hospitalization duration for preterm infants (2). Given that osteopathy focuses on the whole individual, demonstrating its effect on isolated symptoms or localized pathologies is challenging due to multifactorial causes. However, assessing comfort or quality of life is meaningful; in the case of premature newborns, hospitalization duration—linked to their autonomy—is a holistic measure of care effectiveness.

Since 2010, osteopathic treatment (OMT) has been offered in our maternity unit for breastfeeding difficulties unresponsive to usual support methods. In our unit, we conducted two unpublished observational studies: The first study involved the 1.5-month follow-up of 27 mother-infant dyads who received osteopathic care due to a risk of lactation cessation caused by breastfeeding pain or the infant’s inability to latch. At 1.5 months, 25 dyads (92%) were still breastfeeding. Mothers were asked about their experiences and perceptions during and after the osteopathic treatment. Their responses contributed to the questions proposed at 1 month for Amatosteo. The second study focused on the follow-up of 50 dyads who experienced pain rated above 7/10 in at least one breast upon returning home. Among them, 25 dyads (50%) spontaneously decided to receive osteopathic care promptly. At 1 month, 85% of the dyads who underwent osteopathic treatment were still breastfeeding, compared to 15% of those who did not receive such care.

For both observations, at least 85% of mothers were breastfeeding. However, in the first study, there was no comparison group, and in the second study, a major bias stems from the possibility that mothers who seek osteopathic care may have a stronger determination to continue breastfeeding.

These preliminary findings prompted us to evaluate the effectiveness of OMT for mother-newborn dyads using a randomized controlled trial in cases of severe breastfeeding pain unresponsive to standard maternity care.

## 2 Materials and methods

### 2.1 Methodological approach

This is a comparative, randomized, open-label clinical trial. The treatment is administered by one of two osteopathic physicians following a pediatric examination to confirm the absence of contraindications.

### 2.2 Inclusion criteria

Inclusion criteria include painful breastfeeding at one or both breasts (a score >7 on a numeric pain scale ranging from 0 to 10, where 0 is no pain and 10 is the worst pain experienced by the patient) and failure of usual support provided to the mother–child dyad during hospitalization.

Mothers must be adults (≥18 years old) who have chosen either exclusive or mixed breastfeeding, speak and understand French, are affiliated with or beneficiaries of social security, and provide free, informed, written consent (co-signed by the father).

Eligible newborns are singleton infants born at 37 weeks of gestation or later, weighing at least 2,500 grams at birth, and aged at least 36 h.

### 2.3 Exclusion criteria

For mothers: psychiatric disorders.

For newborns: unstabilized infections, respiratory distress requiring assistance for more than 1 h, metabolic disorders requiring intravenous infusion, benign tumors or oncological processes, acute neurological conditions, or congenital malformations preventing normal lactation.

### 2.4 Procedure

#### 2.4.1 Untreated group (control group)

This group receives usual aids to breastfeeding in the postpartum unit (breastfeeding aid equipment, help with the installation of the dyad and the positioning of the baby, help with lactation consultant) and follow-up care at home.

#### 2.4.2 Treated group

The ideal osteopathic treatment involves tactile listening to the patient in their entirety, without prejudice or predefined expectations, by the osteopath maintaining neutrality. The practitioner uses their hands to follow movements sensed in the patient’s body. The mother is comfortably seated semi-upright in her bed, with the baby placed on their back beside her. Occasionally, the baby must be placed on the mother’s chest to achieve calm.

The osteopath’s hands initially hover over the baby’s body before gently making contact (permission to touch is sought). The primary principle of touch involves “receiving” information through the hands (a welcoming touch) rather than seeking it actively (which would be intrusive). The treatment progresses by focusing on areas of tension until a release is felt, often accompanied by the baby repositioning themselves.

At times, the baby may indicate a need to be on their mother’s chest (a distinctive cry), where they find comfort and naturally position themselves with the mother’s support. The mother’s hands typically rest on the baby’s neck and upper back, and pelvis.

The osteopath continues treatment by placing their hands over the mother’s hands, sensing movements in the baby or the mother’s hands. The conclusion of the treatment is marked by a general sense of relief and equilibrium in the baby, mother, and their connection, typically lasting about 40 min.

Afterward, gentle mobilizations of the newborn are performed by the mother, guided by the osteopath, to help her identify a comfortable breastfeeding position that reduces or eliminates pain. Standard postpartum breastfeeding support by midwives and auxiliary nurses continues throughout the hospital stay and afterward at home.

### 2.5 Outcome measures

#### 2.5.1 Primary outcome

The primary outcome is the rate of exclusive or mixed breastfeeding at 1 month postpartum.

#### 2.5.2 Secondary outcomes

- Rate of exclusive breastfeeding at 1 month postpartum.
- Breast pain intensity in each breast.
- Weight of the baby.
- Responses to three closed-ended questions asked to mothers in both groups (treated and control). The questions were:

o Did the treatment have any effect on breastfeeding?
o Did it improve your relationship with your baby?
o Did it enhance your well-being or that of your baby?

### 2.6 Study duration and location

Each dyad participated in the study for 1 month. The study spanned 48 months, from March 23, 2022, to April 23, 2024. All participants were recruited in the postpartum unit at the Aliénor d’Aquitaine maternity hospital, Bordeaux University Hospital.

### 2.7 Randomization

Pain was assessed at least 36 h postpartum. For cases scoring above 7/10, oral and written information about the study was provided to both parents. Upon consent, randomization was performed by a clinical research associate using a dedicated computer located in a separate hospital building.

### 2.8 Statistics

We hypothesize that, in the control group (without osteopathic care), the proportion of breastfeeding (exclusive or not) at 1 month postpartum is 50%, while it is 85% in the treatment group (with at least one osteopathic consultation). Indeed, an internal study at the CHU of Bordeaux showed that for postpartum women experiencing breastfeeding pain that was resistant to treatment, an osteopathic consultation improved the proportion of women breastfeeding at 1.5 months postpartum: 85% versus 15%. Given that half of the patients will consult an osteopath afterward, this corresponds to a breastfeeding proportion at 1 month of 50% = (15% + 85%) / 2.

Thus, with a Type I error risk (*α*) of 5% and a power of 90%, accounting for 10% loss to follow-up, the number of patients to be included in each group was estimated at 40, or 80 patients in total (SAS® version n°9.4, “Proc power” with a Pearson Chi-square test).

Statistical analysis was performed using SAS® software, version 9.4. Breastfeeding rates were analyzed using Fisher’s exact test. Quantitative variables that did not follow a normal distribution were described as medians with interquartile ranges (25th and 75th percentiles) and compared using the Wilcoxon test. A significance level of 5% was applied.

## 3 Results

### 3.1 Population

**Figure 1:**
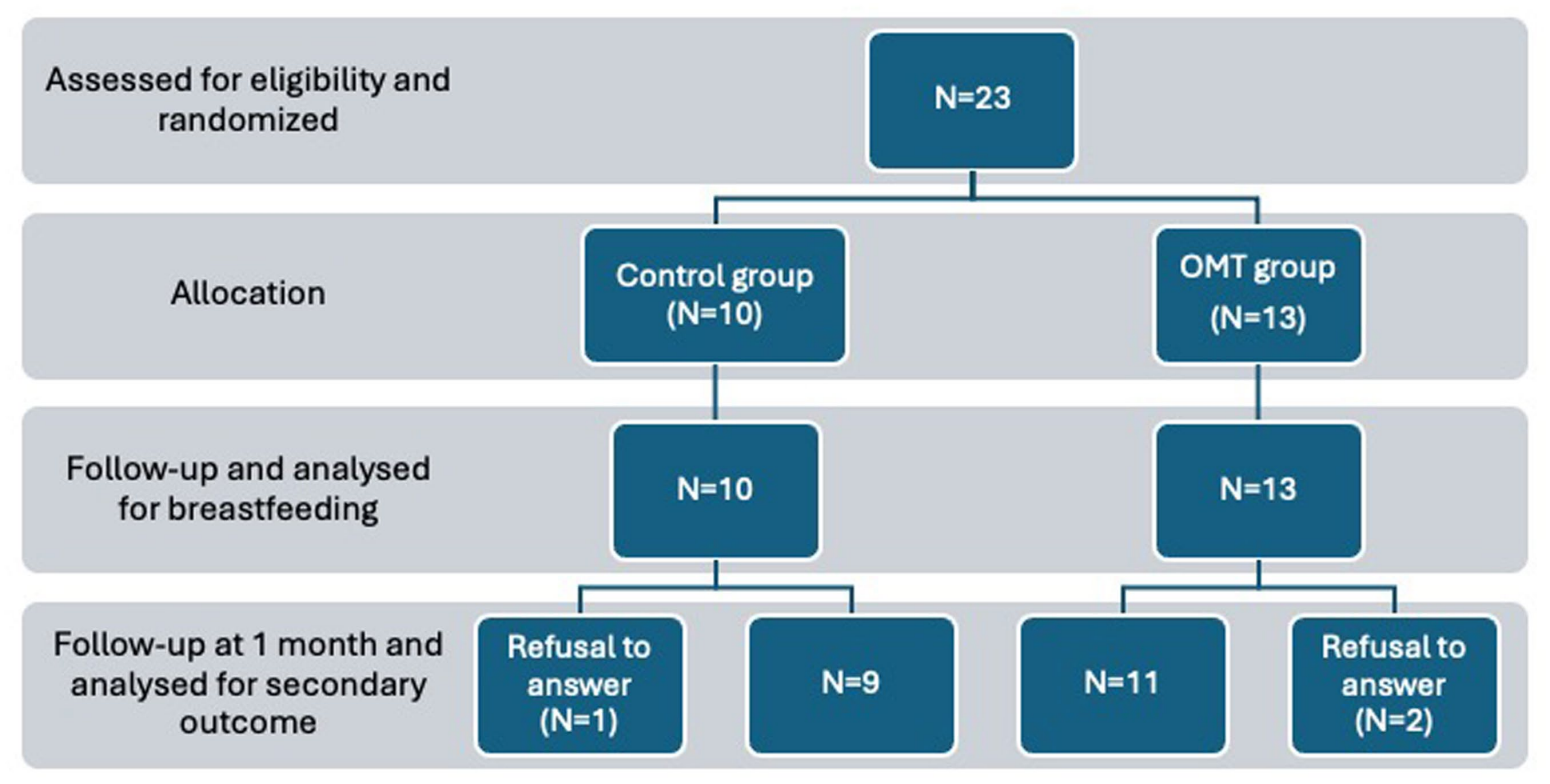
Flow chart of the study.

There are three incomplete follow-ups for which the babies’ weights and responses to the questions are missing. Three mothers stopped breastfeeding very early and refused to respond to follow-up questions at 1 month: two in the treated group and one in the control group. Weight measurements were missing for two infants in the untreated group and three in the treated group (Figure 1). However, we have all the data regarding the primary objective and the first secondary objective.

### 3.2 Comparability of both groups

There were no differences between the groups regarding maternal age, number of previous pregnancies, or previous breastfeeding experiences (in both groups, only two mothers had breastfed for more than 1 month). Medical events during pregnancy that could have disrupted outcomes (e.g., positive trisomy 21 screening, hemorrhagic episodes, preterm labor threats, premature rupture of membranes, gestational diabetes, or preeclampsia) were comparable between the groups (Table 1).

**Table 1:** Pregnancy and delivery context.

Pregnancy and delivery context	Control (*N* = 10)	OMT (*N* = 13)	*p*-value
Maternal age (years)	30.5 (26.5–34.7)	33.0 (29.5–36.5)	0.88
Number of previously breastfed babies	2 (20%)	5 (38%)	0.40
Cumulative lactation duration (weeks)	0 (0–6.75)	0 (0–5.50)	0.60
Positive trisomy 21 screening	1 (10%)	1 (7.7%)	1.00
Amniocentesis	1 (10%)	0	0.43
Ultrasound anomaly	0	2 (15%)	0.48
Maternal pathology	1 (10%)	3 (23%)	0.60
Hemorrhagic processes	1 (10%)	2 (15%)	1.00
Threatened preterm labor	0	1 (7.7%)	1.00
Premature rupture of membranes	0	1 (7.7%)	1.00
Gestational age (weeks)	39 (39–40.7)	39 (39–40)	0.37
Induced labor	3 (30%)	3 (23%)	1.00
Epidural anesthesia	10 (100%)	11 (85%)	0.48
Apgar score at 1 min	9.5 (8.25–10)	10 (10–10)	0.21
Apgar score at 5 min	10 (10–10)	10 (10–10)	0.56
Resuscitation maneuvers	0	0	1.00
Respiratory distress	0	1 (7.7%)	1.00
Birth weight (grams)	3,450 (3,205–3782.5)	3,630 (3,380–3,775)	0.37
Highest pain score	10 (9–10)	9 (8.5–10)	0.48

Values in parentheses represent medians with interquartile ranges (25 and 75%).

No differences were observed regarding the initiation of labor (spontaneous or induced) or the use of epidural anesthesia.

Similarly, there was no significant difference in the highest reported breast pain scores.

For newborns, there were no differences in Apgar scores at 1 and 5 min, birth weight, or need for resuscitation. One transient respiratory distress case occurred in the treated group: this respiratory distress resolved quickly and had no impact on lactation.

### 3.3 Breastfeeding rates at 1 month

At 1 month postpartum (Table 2), 11 out of 13 mothers in the treated group were still breastfeeding (84.6%), compared to 3 out of 10 mothers in the control group (30%). The difference was statistically significant (*p* = 0.01).

**Table 2:** Results.

Results at 1 Month	Control (*N* = 10)	Treatment (*N* = 13)	*p*-value
Breastfeeding (Yes)	3 (30%)	11 (84.6%)	0.01
Exclusive breastfeeding	1 (10%)	8 (61.5%)	0.03
Baby weight (grams)	4230 (3,880–4,440) *N* = 8	4430 (4,375–4,575) *N* = 10	0.06

Baby weight is presented as the median, with interquartile ranges (25 and 75%) in parentheses.

Exclusive breastfeeding rates at 1 month were 61.5% in the treated group (8 out of 13 mothers) compared to 10% in the control group (1 out of 10 mothers), with a significant difference (*p* = 0.03).

### 3.4 Breastfeeding pain

In the control group, 2 out of 3 mothers continued to experience pain during breastfeeding (maximum pain scores of 3 and 6). In the treated group, 4 out of 11 mothers reported pain (maximum scores of 2, 2, 3, and 3).

### 3.5 Infant weight

The median weight at 1 month was 4,230 grams in the control group and 4,430 grams in the treated group (Table 2). The difference was not statistically significant (*p* = 0.06).

### 3.6 Maternal feedback on osteopathic treatment

When asked whether the treatment had any effect on breastfeeding, 10 of the 11 mothers in the treated group who were still breastfeeding at 1 month believed the session had a positive effect.

Among the 3 mothers in the control group whose infants received osteopathic treatment after discharge, 2 believed the treatment had a positive impact and were still breastfeeding at 1 month. One mother believed the treatment had no effect and had ceased breastfeeding by 1 month.

To the questions “Did the treatment improve your relationship with your baby?” and “Did the treatment improve your well-being or your baby’s?,” all 11 treated mothers and the 2 untreated mothers who consulted an osteopath answered positively.

## 4 Discussion

### 4.1 Methodology

Offering joint treatment to both mother and infant stems from clinical observations gathered over a decade of osteopathic care in our maternity unit. Treating the baby in isolation almost systematically leads to failure. Challenges frequently involve both the mother and the baby. Treating both partners facilitates the synchronization needed to optimize breastfeeding.

This approach aligns with the holistic support of breastfeeding recently conceptualized under the term “Gestalt Breastfeeding” (7), which acknowledges the interactive nature of the process, with the mother being encouraged to support the regulation of her baby’s state by reading and responding to the infant’s cues.

### 4.2 Inclusion criteria

The stringent inclusion criterion—a pain score of 8 or higher—was chosen to address the emergency to improve breastfeeding rates. Severe pain often leads to rapid, irreversible decisions to supplement with formula or discontinue breastfeeding. This emergency also made the impact of osteopathic treatment easily observable and measurable.

Preliminary evaluations indicated that at least two mothers per week experienced this level of pain (score 8, 9, or 10) in one or more breasts. However, recruitment was challenging because osteopathy is widely recognized and used routinely in France. As a result, some mothers refused participation due to unwillingness to be randomized. Conversely, other mothers were adamantly opposed to osteopathy. Furthermore, in many cases, the decision to stop breastfeeding had already been made—especially when the inclusion in the study was proposed more than 48 h postpartum.

### 4.3 Results

#### 4.3.1 Breastfeeding rates

Despite the small sample size, breastfeeding rates were significantly improved with OMT. The observed rate of 85% in the treated group aligns with expectations. In the control group, it was anticipated that 50% of mothers would consult an osteopath after discharge. In fact, 3 mothers sought osteopathic care, 2 of whom continued breastfeeding at 1 month. The mother practicing exclusive breastfeeding had received two osteopathic treatments within the first month.

A similar randomized trial, Neosteo (8), conducted in Nantes, France, included three groups: a placebo group, an untreated group, and a treated group, each comprising 64 dyads. The inclusion criterion is based on a composite score of breastfeeding difficulties (IBFAT). The primary endpoint was breastfeeding rates at 1 month. No significant differences were observed between the groups. However, in this study, osteopathic treatment was administered to the baby separated from the mother, who was positioned behind a screen at a distance. We believe that the osteopathic treatment being performed on the baby in isolation from the mother contributed to the ineffectiveness of the therapeutic intervention. For this reason, in our study, we ensured the continuation of our usual osteopathic care, focusing on the mother-baby dyad and their osteopathic interaction, considering painful breastfeeding as an indicator or alarm signal of an overall imbalance, with its resolution indicating the restoration of mother-baby equilibrium. The choice of a composite criterion leads to more varied causes underlying the disorder, making it challenging to demonstrate a difference.

A Canadian study (3) compared, without blinding, standard breastfeeding support versus standard breastfeeding support combined with osteopathic care: among 97 newborns in two balanced groups, a significant difference was observed based on another composite score of breastfeeding efficiency (LATCH), which was also the inclusion criterion.

Two literature reviews, from 2019 (9) and 2022 (10) respectively, found very few randomized studies conducted with robust scientific criteria to evaluate osteopathy, concluding a lack of evidence for the efficacy of osteopathy in children, except in the context of prematurity (2). This is why we conducted this study, albeit with a small sample size.

#### 4.3.2 Infant weight

The average weight gain was higher in the treated group compared to the control group. The difference was not statistically significant (*p* = 0.06). This suggests that the infants in the treated group have sufficient milk intake and that, therefore, breastfeeding is effective. Specifically, the average weight gain was 875 grams in the treated group and 679 grams in the untreated group.

#### 4.3.3 Osteopathic treatment

Firstly, the term “osteopathic manipulative treatment” in the literature encompasses various techniques, sometimes even including chiropractic practices. Osteopathy involves numerous techniques, such as high-velocity low-amplitude (HVLA) manipulation, myofascial release (MFR), cranial techniques, muscle energy techniques, and counterstrain. Myofascial release techniques are more appropriate for newborns. These distinctions are rarely detailed in publications and may explain the variability in reported outcomes.

Secondly, when treating newborns—particularly in the context of breastfeeding—osteopathic care in the absence of the mother is unlikely to be effective. This is especially true since successful treatment requires a calm and relaxed state, which is rare for a baby separated from their mother.

The strength of our study lies in its detailed description of the osteopathic technique we have been practicing for two decades but never formalized. We hope other teams will adopt this holistic approach, whose benefits extend far beyond breastfeeding, fostering parent-infant connections, as previously described in our earlier reports.

### 4.4 Limitations

#### 4.4.1 Sample size

The study duration was extended by 12 months due to recruitment difficulties. Despite this extension, the study was eventually halted due to team constraints. However, there was no loss to follow-up for the primary outcome. Three mothers declined further participation after reporting they had ceased breastfeeding.

Only 30% of untreated dyads consulted an osteopath, with only one session being provided to the infant. Additionally, the pain score in the untreated group remains high, leading to earlier cessation of breastfeeding and making it unlikely that osteopathic care would have been pursued in time to make a difference.

We did not provide any instructions to the mothers in the untreated group (neither encouragement nor prohibition). Our statisticians did not find it appropriate to consider patients who received treatment after discharge as part of the treated group. This is also why the required sample size was high, as it accounted for the 50% probability of seeking osteopathic care upon returning home and before the 1-month evaluation.

These considerations, along with the extension from 12 to 24 months for participant inclusion, justified the study’s conclusion.

#### 4.4.2 Lack of blinding and placebo

Blinding was not feasible because it is impossible to treat the mother and newborn together while maintaining blinding. Furthermore, osteopathy is widely recognized today, and participants would immediately identify a placebo intervention.

Administering a placebo to newborns also poses challenges, as they lack the ability to anticipate or comprehend what is being offered. A randomized study (11) compared standard care to standard care plus placebo osteopathic treatment in hospitalized preterm infants, finding no differences. Similarly, results from the Neosteo study (8) showed no difference between standard care and standard care plus placebo osteopathic treatment.

Moreover, the involvement of midwives and lactation consultants in positioning the baby may act as a placebo in its own right. Many painful situations are alleviated by their guidance. When these methods fail—one of the study’s inclusion criteria—more precise or subtle adjustments, accessible through osteopathy, may be required.

This situation, perceived as a failure by the healthcare team supporting breastfeeding, is quickly reversed after OMT (Osteopathic Manipulative Treatment): breastfeeding continues because the team regains its usual efficiency and can once again support the dyad. The osteopath is, in fact, integrated into the team to strengthen it, not replace it. This is indeed complementary medicine as defined by the French Ministry of Health.

### 4.5 Perspectives

The small sample size, despite significant results, indicates the need for further studies to confirm these findings. Based on recruitment challenges, we propose offering osteopathic care as soon as pain scores are very high despite standard interventions. Concurrent treatment of the mother and baby is essential. The osteopathic technique should involve gentle, non-invasive methods (“soft touch”) applied to either the newborn or the mother.

The refusal of randomization by many participants could be addressed in future studies by employing a crossover design, randomizing the timing of treatment (e.g., immediate versus 15 or 30 days later), or using Zelen’s design (12, 13). This design is suited to studies where the intervention is already widely accepted, such as osteopathy, and where untreated participants may perceive a lack of fairness. In Zelen’s approach, participants are first included based on disease monitoring, and randomization occurs later, with differing information sheets for treated and untreated groups. While this design could address some challenges, it is difficult to implement under current French research regulations and ethical committee standards.

## 5 Conclusion

This comparative, randomized, open-label study conducted in a type 3 maternity hospital formalized our 15 years of clinical observation of the benefits of early osteopathic treatment for mother-baby dyads experiencing severe breastfeeding pain. We targeted dyads at the highest risk of breastfeeding cessation using strict inclusion criteria for intense pain (scores of 8, 9, or 10) in at least one breast during feeding, despite support from a team trained in breastfeeding assistance. After explaining the study and randomization process, only randomized dyads received osteopathic treatment.

The results demonstrate that without OMT, even highly motivated mothers often discontinue breastfeeding (with a continuation rate of only 30% at 1 month). By contrast, most mothers receiving OMT continued their breastfeeding plans, with a continuation rate of nearly 85% at 1 month.

The distinctive feature of this study lies in the holistic osteopathic care we describe, based on extensive experience and integrating the physiological specificities of both the newborn and the mother. This approach emphasizes the need to reconnect them for optimal outcomes.

Finally, initially challenged by its inability to find a solution to the initial pain, the healthcare team, after osteopathic care, can regain its effectiveness in providing breastfeeding support, which is essential for its continuation. In this context, osteopathy serves as a boost to overcome a challenge and allows healthcare teams to optimize their capabilities.

## Data Availability

The raw data supporting the conclusions of this article will be made available by the authors, without undue reservation.
